# DeepProtein: deep learning library and benchmark for protein sequence learning

**DOI:** 10.1093/bioinformatics/btaf165

**Published:** 2025-05-19

**Authors:** Jiaqing Xie, Yuqiang Li, Tianfan Fu

**Affiliations:** Computer Science Department, ETH Zurich, Zürich 8006, Switzerland; AI for Science, Shanghai Artificial Intelligence Laboratory, Shanghai 200030, The People’s Republic of China; State Key Laboratory for Novel Software Technology at Nanjing University, School of Computer Science, Nanjing University, Nanjing, Jiangsu 210023, China

## Abstract

**Motivation:**

Deep learning has deeply influenced protein science, enabling breakthroughs in predicting protein properties, higher-order structures, and molecular interactions.

**Results:**

This article introduces DeepProtein, a comprehensive and user-friendly deep learning library tailored for protein-related tasks. It enables researchers to seamlessly address protein data with cutting-edge deep learning models. To assess model performance, we establish a benchmark that evaluates different deep learning architectures across multiple protein-related tasks, including protein function prediction, subcellular localization prediction, protein–protein interaction prediction, and protein structure prediction. Furthermore, we introduce DeepProt-T5, a series of fine-tuned Prot-T5-based models that achieve state-of-the-art performance on four benchmark tasks, while demonstrating competitive results on six of others. Comprehensive documentation and tutorials are available which could ensure accessibility and support reproducibility.

**Availability and implementation:**

Built upon the widely used drug discovery library DeepPurpose, DeepProtein is publicly available at https://github.com/jiaqingxie/DeepProtein.

## 1 Introduction

Understanding the representation of proteomics is vital in developing traditional biological and medical progress (Wu *et al.* 2022b, Fu *et al.* 2024), multi-omics genomics (Chen *et al.* 2021, Wu *et al.* 2022a), and curing human diseases (Chen *et al.* 2024a,c). Being the working house of the cell, it provides many functions that support human daily life, such as catalyzing biochemical reactions that occur in the body as a role of enzymes and providing helpful immune responses against harmful substances that act as immunoglobulin (Wu *et al.* 2024). Under the necessity of analyzing those useful proteins, several related protein databases are available to researchers (Bairoch and Apweiler 2000, Helen *et al.* 2000, Pontén *et al.* 2008, Consortium 2015). Apart from the 2D database, some recent 3D Protein Database used AlphaFold 2.0 (Jumper *et al.* 2021) is important to better assist in learning those representations in 3D space. The success of AlphaFold 2.0 has sparked a significant increase in interest in using machine learning techniques for protein learning tasks, of which the goal is to improve our understanding of proteins’ biochemical mechanisms.

Deep learning has revolutionized protein science, driving significant advancements in various protein-related tasks. These include protein–protein interactions (PPIs) (Gainza *et al.* 2020), protein folding (Chen *et al.* 2016, Dimitra *et al.* 2020, Jumper *et al.* 2021, Lu 2022), protein–ligand interactions (Li *et al.* 2021a, Xia *et al.* 2023), and protein function and property prediction (Gligorijević *et al.* 2021, Sevgen *et al.* 2023). The development of deep neural architectures has played a crucial role in these tasks, with approaches leveraging both *sequence-based* and *structure-based* models. *Sequence-based* models, such as convolutional neural networks (CNNs) (Shanehsazzadeh *et al.* 2020) and transformers, have shown strong performance in protein learning tasks. The TAPE Transformer (Rao *et al.* 2019) and pre-trained transformer models such as ProtBERT (Brandes *et al.* 2022) have demonstrated the effectiveness of self-supervised learning in capturing protein sequence representations. Beyond sequence-based methods, *structure-based* deep learning has gained traction with graph neural networks (GNNs), which leverage 3D structural information to enhance structural property (Jing *et al.* 2021, Zhang *et al.* 2022). Recently, graph transformers have emerged as a powerful alternative, combining the advantages of transformers (global attention) and message-passing neural networks (sparse attention) to model protein structures more effectively (Yuan *et al.* 2022, Gu *et al.* 2023).

While transformers have been considered state-of-the-art in previous benchmarks (Xu *et al.* 2022), comprehensive comparisons between CNN, transformer, GNN, and other advanced architectures remain under-explored. This gap motivates us to systematically integrate and evaluate these methods in our benchmark. Furthermore, pretraining strategies have been prevailing in protein science, which have utilized the large-scale unlabeled protein data to improve downstream performance (Lu *et al.* 2024, Yue *et al.* 2024). With the advent of large foundation models, protein properties can now be inferred through prompt engineering, such as BioMistral (Labrak *et al.* 2024), BioT5/BioT5+ (Pei *et al.* 2023, 2024), and ChemLLM (Zhang *et al.* 2024). Both advancements in protein pretraining and question-answering language models in protein brought more possibilities to the field of protein engineering.

*Challenges.* Previous benchmarks related to molecular learning have offered valuable insights regarding their respective libraries and implementation interfaces. DeepPurpose (https://github.com/kexinhuang12345/DeepPurpose) (Huang *et al.* 2021a,b) has provided an interface that implements the task with a majority of drug discovery tasks, which only has PPI and protein function prediction implemented. Datasets on proteins are also lacking. TorchProtein (https://github.com/DeepGraphLearning/PEER_Benchmark) (Xu *et al.* 2022), also named as PEER, implemented most of the tasks in the protein field. In terms of models, the focus has largely been on *sequence-based* methods: Convolutional Neural Networks (CNNs), Transformers, and ESM architectures. This suggests that there are still many *structure-based* methods (GNN) or pre-trained protein language models available (such as ProtBert or Prot-T5) for consideration. Furthermore, PEER’s interface is not user-friendly without prior domain knowledge in graphs and biochemistry. This presents an opportunity to improve the existing interface regarding simplicity and comprehensibility.

*Solutions.* To address these challenges, in this article, we propose DeepProtein, which aims to benchmark mainstream and cutting-edge deep learning models on a wide range of AI-solvable protein sequence learning tasks. We investigate the performance of various deep learning models on a wide range of protein sequence learning tasks. We analyze each method’s advantages and disadvantages when performing each task (working as the explainer for each task). We have provided user-friendly and well-wrapped interfaces to facilitate domain experts’ research.

*Contribution.* Our key contributions are summarized as:

- **Comprehensive benchmarking**: We curate a benchmark to evaluate the performance of eight coarse-grained deep learning architectures, including CNNs, CNN-RNNs, RNNs, transformers, graph neural networks, graph transformers, pre-trained protein language models, and large language models. This benchmark covers eight protein learning tasks, including protein function prediction, protein localization prediction, PPI prediction, antigen epitope prediction, antibody paratope prediction, CRISPR repair outcome prediction, antibody developability prediction, and protein structure prediction. Our benchmark demonstrates the strength, scalability and limitation of the mentioned approach respectively.
- **User-friendly library**: We develop DeepProtein, a specialized deep learning library that integrates these neural network architectures for protein-related tasks. DeepProtein offers a simple, command-line interface for running models on all supported tasks, making it accessible to researchers with minimal deep learning expertise.
- **Enhanced accessibility**: We provide comprehensive documentation, tutorials, and pre-configured pipelines. Inherited from DeepPurpose (Huang *et al.* 2021a,b), our library ensures seamless integration with existing protein frameworks or personalized protein databases, and enables reproducibility.
- **Fine-tuned models—DeepProt-T5**: We have released our fine-tuned Prot-T5-XL models for each task, which is available on Hugging Face. The model family is called DeepProt-T5. These models achieve either state-of-the-art or competitive performance across our DeepProtein benchmark, so there’s no need for the redundant retraining process, making model deployment much more efficient and convenient.

### 1.1 Related works

Benchmarks and libraries are crucial in AI-based therapeutic science, e.g. multi-omics data (Lu 2018), protein learning (Xu *et al.* 2022), small-molecule drug discovery (Gao *et al.* 2022, Xu *et al.* 2024), and drug development (clinical trial) (Chen *et al.* 2024b, Wang *et al.* 2024). They provide standardized metrics for evaluating the performance of various algorithms and models. These benchmarks enable researchers to compare different approaches systematically, ensuring reproducibility and reliability of results.

**Figure 1.**
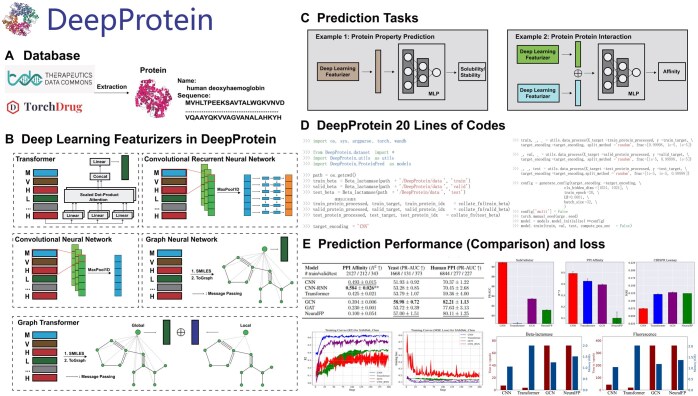
DeepProtein framework. Part 1. (A) DeepProtein is mainly selected from TorchDrug and Therapeutics Data Commons (TDC), where only protein tasks are considered, and all drug-related tasks are excluded, such as drug–target interactions. Specifically, DeepPurpose has established such a pipeline in their library. (B) Both sequence-based and structure-based methods are included in DeepProtein. For some graph neural networks, we utilized edge featurizers to generate additional edge information since inputs are 2D. Protein language models, large language models, and our pre-trained T5 (DeepProt-T5) are discussed in Figure 2. (C) Task types are: protein function prediction, subcellular localization prediction, PPI prediction, and protein structure prediction. They can be classified as either a 1 (protein)-to-1 (aim) problem or a 2 (proteins)-to-1 (aim) problem, which meets the researchers’ needs. (D) An earlier version of DeepProtein could be executed within 20 lines of code. The newest version of DeepProtein could be executed within 10 lines of code, where we further wrapped the data processing steps. Researchers can also provide their own data to either train or perform inference with the help of DeepProtein. (E) In this article, we provide comprehensive results, including the performance of each model on corresponding tasks, the differences among sequence-based models, structure-based models, and pre-trained protein language models, and the computation resources, including time-stamps and GPU memory assumptions. As DeepProtein supports wandb, we also provide two wandb repositories that record the results of all experiments, which are https://wandb.ai/jiaqing/DeepProtein? nw=nwuserjiaqing and https://wandb.ai/jiaqing/DeepPurposePP. Tables and figures are presented later in this article.

In this section, we briefly discuss the benchmark studies in this area. Proteins are vital in drug discovery because they often serve as the primary targets for therapeutic agents, influencing disease mechanisms and biological pathways. Additionally, proteins play key roles in various cellular processes, making them essential for identifying potential drug candidates and biomarkers in the drug development pipeline. A couple of protein learning benchmarks are developed, including PEER (Xu *et al.* 2022), DeepPurpose (Huang *et al.* 2021a,b), FLIP (Dallago *et al.* 2021), TAPE (Rao *et al.* 2019). Table 1 compares DeepProtein with existing AI-based protein learning benchmarks. We extend the scope of existing protein learning benchmarks by incorporating more protein learning datasets, more cutting-edge deep learning models, and enhancing user-friendliness (Fig. 1).

**Table 1.** Comparison of benchmark studies on protein sequence learning.^a^

Datasets	DeepPurpose	FLIP	TAPE	PEER	TDC (data only)	DeepProtein
References	Huang *et al.* (2021)	Dallago *et al.* (2021)	Rao *et al.* (2019)	Xu *et al.* (2022)	Huang *et al.* (2021)	ours
Fluorescence	×	√	√	√	×	√
β -lactamase	×	×	×	√	×	√
Solubility	×	×	×	√	×	√
Stability	×	√	√	√	×	√
Subcellular (binary)	×	×	×	√	×	√
PPI affinity	×	×	×	√	×	√
Yeast PPI	×	×	×	√	×	√
Human PPI	×	×	×	√	×	√
IEDB	×	×	×	×	⋄	√
PDB-Jespersen	×	×	×	×	⋄	√
SAbDab-Liberis	×	×	×	×	⋄	√
TAP	×	×	×	×	⋄	√
SAbDab-Chen	×	×	×	×	⋄	√
CRISPR-Leenay	×	×	×	×	⋄	√
Fold	×	×	×	√	×	√
Secondary Structure	×	×	√	√	×	√

a TDC provides AI-ready datasets but does not contain protein learning benchmarks (denoted ⋄).

## 2 Methods: DeepProtein library and benchmark

### 2.1 AI-solvable protein problems

In this section, we elaborate on a couple of AI-solvable protein problems and the related datasets.

- **Protein function prediction**. Protein function prediction involves determining the biological roles and activities of proteins based on their sequences or structures. This process is crucial for understanding cellular mechanisms and interactions, as a protein’s function is often linked to its sequence composition and the context of its cellular environment. Machine learning algorithms are used to analyze known protein databases, identifying patterns and features that correlate with specific functions. Accurate predictions can facilitate drug discovery, help elucidate disease mechanisms, and support advancements in synthetic biology by providing insights into how proteins can be engineered for desired activities (Zhang *et al.* 2021). We consider the following datasets.
  - **Fluorescence** (Sarkisyan *et al.* 2016). Protein fluorescence refers to the phenomenon where certain proteins can emit light of a specific wavelength when excited by light of a shorter wavelength. It is a widely used technique to study protein structure, dynamics, interactions, and function. The dataset consists of 54 025 protein sequences with real-valued groundtruth. The label is the logarithm of fluorescence intensity.
  - **Stability** (Rocklin *et al.* 2017). Protein stability is the capacity of a protein to preserve its 3D structure and functional characteristics across different environmental conditions. This stability is essential for the proper functioning and longevity of proteins within biological systems. A protein’s stability is influenced by its ability to withstand denaturation, aggregation, and degradation. The dataset comprises 68 934 protein sequences with real-valued groundtruth.
  -
β -**lactamase** (Gray *et al.* 2018). This task aims to predict the increased activity of β-lactamase, the most common enzyme that provides gram-negative bacteria with resistance to beta-lactam antibiotics through single mutations. The dataset consists of 5198 protein sequences with real-valued groundtruth. The groundtruth refers to the experimentally determined fitness score, which measures the scaled mutation effect for each mutant.
  - **Solubility** (Khurana *et al.* 2018). Protein solubility is the capacity of a protein to dissolve or remain dispersed in a solution. This property is crucial for determining how the protein behaves and functions in various biological and industrial contexts. Several factors influence a protein’s solubility, including its amino acid composition, ionic strength, pH, temperature, and the presence of other molecules in the solution. The dataset consists of 71 419 protein sequences with binary labels.
- **Protein localization prediction**. Accurate localization predictions can enhance drug development by informing target identification and improving therapeutic efficacy, particularly in treating diseases linked to protein mislocalization. Additionally, insights gained from localization predictions facilitate the mapping of biological pathways, aiding in the identification of new therapeutic targets and potential disease mechanisms.
  - **Subcellular** (Almagro Armenteros *et al.* 2017). The task predicts the location of a natural protein within the cell. The dataset consists of 13 961 data samples with categorical labels (10 classes, {0,1,2,…,9}).
  - **Binary** (Almagro Armenteros *et al.* 2017). It is a simpler version of the previous task (10-category classification), where the model is trained to roughly forecast each protein as either “membrane-bound” or “soluble” (i.e. binary classification). The dataset comprises 8634 data samples with binary labels.
- **PPI.** Proteins are the essential functional units in human biology, but they seldom operate in isolation; rather, they typically interact with one another to perform various functions. Understanding PPIs is crucial for identifying potential therapeutic targets for disease treatment. Traditionally, determining PPI activity requires costly and time-consuming wet-lab experiments. PPI prediction seeks to forecast the activity of these interactions based on the amino acid sequences of paired proteins.
  - **PPI Affinity** (Iain *et al.* 2012). It consists of 2682 protein–protein pairs with real-valued groundtruth.
  - **Yeast** (Guo *et al.* 2008). The dataset comprises 2172 protein–protein pairs with binary labels.
  - **Human PPI** (Pan *et al.* 2010). The dataset comprises 7348 protein–protein pairs with binary labels.
- **Epitope prediction**. An epitope, also known as an antigenic determinant, is the region of a pathogen that can be recognized by antibodies and cause an adaptive immune response. The epitope prediction task is to distinguish the active and nonactive sites from the antigen protein sequences. Identifying the potential epitope is of primary importance in many clinical and biotechnologies, such as vaccine design and antibody development, and for our general understanding of the immune system (Du *et al.* 2024). In epitope prediction, the machine learning model makes a binary prediction for each amino acid residue. This is also known as ***residue-level classification***.
  - **Immune epitope database (IEDB)** (Vita *et al.* 2019). It consists of 3159 antigens with binary labels on each amino acid. The label indicates whether the amino acid belongs to the epitope, i.e. active position in binding. It can be downloaded from TDC (https://tdcommons.ai/single_pred_tasks/epitope/).
  - **PDB-Jespersen** (Jespersen *et al.* 2017). It consists of 447 antigens with binary labels on each amino acid. It is curated by (Jespersen *et al.* 2017) and is extracted from PDB (Protein Data Bank). It can be downloaded from TDC (https://tdcommons.ai/single_pred_tasks/epitope/).
- **Paratope prediction**. Antibodies, or immunoglobulins, are large, Y-shaped proteins that can recognize and neutralize specific molecules on pathogens, known as antigens. They are crucial components of the immune system and serve as valuable tools in research and diagnostics. The paratope, also referred to as the antigen-binding site, is the region that specifically binds to the epitope. While we have a general understanding of the hypervariable regions responsible for this binding, accurately identifying the specific amino acids involved remains a challenge. This task focuses on predicting which amino acids occupy the active positions of the antibody that interact with the antigen. In paratope prediction, the machine learning model makes a binary prediction for each amino acid residue. This is also known as ***residue-level classification***.
  - **SAbDab-Liberis** (Liberis *et al.* 2018) is curated from SAbDab (Dunbar *et al.* 2014). It consists of 1023 antibody chain sequences; each antibody contains both heavy and light chain sequences. It can be downloaded from TDC (https://tdcommons.ai/single_pred_tasks/paratope/#sabdab-liberis-et-al).
- **Antibody developability prediction**. Immunogenicity, instability, self-association, high viscosity, polyspecificity, and poor expression can hinder an antibody from being developed as a therapeutic agent, making early identification of these issues crucial. The goal of antibody developability prediction is to predict an antibody’s developability from its amino acid sequences. A fast and reliable developability predictor can streamline antibody development by minimizing the need for wet lab experiments, alerting chemists to potential efficacy and safety concerns, and guiding necessary modifications. While previous methods have used 3D structures to create accurate developability indices, acquiring 3D information is costly. Therefore, a machine learning approach that calculates developability based solely on sequence data is highly advantageous.
  - **TAP** (Raybould *et al.* 2019). It contains 242 antibodies with real-valued groundtruth. Given the sequences of the antibody’s heavy and light chains, we need to predict its developability (continuous value). The input consists of a list containing two sequences: the first representing the heavy chain and the second representing the light chain. It can be downloaded from TDC (https://tdcommons.ai/single_pred_tasks/develop/).
  - **SAbDab-Chen** (Chen *et al.* 2020). It consists of 2409 antibodies with real-valued groundtruth. It is extracted from SAbDab (the structural antibody database) (It is publicly available http://opig.stats.ox.ac.uk/webapps/newSAbDab/SAbDab/.), which is a database containing all the antibody structures available in the PDB (Protein Data Bank), annotated and presented in a consistent fashion (Dunbar *et al.* 2014). Given the antibody’s heavy chain and light chain sequence, predict its developability (binary label). It can be downloaded from TDC (https://tdcommons.ai/single_pred_tasks/develop/).
- **CRISPR repair outcome prediction**. CRISPR-Cas9 is a gene editing technology that allows for the precise deletion or modification of specific DNA regions within an organism. It operates by utilizing a custom-designed guide RNA that binds to a target site upstream, which results in a double-stranded DNA break facilitated by the Cas9 enzyme. The cell responds by activating DNA repair mechanisms, such as nonhomologous end joining, leading to a range of gene insertion or deletion mutations (indels) of varying lengths and frequencies. This task aims to predict the outcomes of these repair processes based on the DNA sequence. Gene editing marks a significant advancement in the treatment of challenging diseases that conventional therapies struggle to address, as demonstrated by the FDA’s recent approval of gene-edited T-cells for the treatment of acute lymphoblastic leukemia. Since many human genetic variants linked to diseases arise from insertions and deletions, accurately predicting gene editing outcomes is essential for ensuring treatment effectiveness and reducing the risk of unintended pathogenic mutations.
  - **CRISPR-Leenay** (Leenay *et al.* 2019). The dataset comprises 1521 DNA sequences (including guide RNA and PAM) with five measured repair outcomes, assessed across various donor populations of primary T cells. It can be downloaded from TDC (https://tdcommons.ai/single_pred_tasks/CRISPROutcome/).
- **Protein structure prediction.** Protein structure prediction (PSP) is a fundamental problem in computational biology, aiming to determine the 3D structure of a protein from its amino acid sequence. Specifically, in our benchmark, the task is to predict the family of a folding or secondary structure family that it belongs to. Since a protein’s structure dictates its function, accurate prediction is crucial for understanding the topology of the protein. PSP can be broadly divided into global topology prediction and local structural prediction, which include tasks such as fold classification and secondary structure prediction:
  - **Fold** (Hou *et al.* 2018). This task involves predicting the global structural topology of a protein and categorizing it into one of the predefined fold classes (within 0, 1, …, 1194). During inference, we predict the class of each protein’s superfamily. It contains 13766 samples overall.
  - **Secondary structure** (Klausen *et al.* 2019). This task focuses on predicting the local structural elements (coil, strand, helix) of each residue in a protein sequence. It serves as an intermediate step for more complex structure prediction tasks and is useful in applications such as functional analysis and multiple sequence alignment. This is a residue-level 3-class classification problem where the number of samples is equal to 11 361.

In this library, we follow the train-validation-test split in PEER benchmark (Xu *et al.* 2022) and TDC (Huang *et al.* 2022). Each individual split is reported from Tables 2–7.

**Table 2.** Results of protein function prediction.^a^

Model	Fluorescence		Stability		β-lactamase		Solubility	
	ρ ↑	MAE ↓	ρ ↑	MAE ↓	ρ ↑	MAE ↓	PR-AUC ↑	Averaged F1 ↑
# train/valid/test	21446/5362/27217		53571/2512/12851		4158/520/520		62478/6942/1999	
CNN	**0.680** ± **0.001**	**0.194** ± **0.001**	0.715 ± 0.025	0.312 ± 0.008	0.721 ± 0.020	0.152 ± 0.001	74.61±0.55	62.50 ± 2.69
CNN-RNN	0.678±0.001	0.268 ± 0.002	0.635±0.025	0.323 ± 0.001	0.695±0.012	0.157 ± 0.002	75.46±0.03	60.87 ± 3.19
Transformer	0.648±0.001	0.371 ± 0.005	0.375±0.052	0.411 ± 0.026	0.310±0.041	0.232 ± 0.007	**78.86** ± **0.46**	61.28 ± 1.42

GCN	0.397±0.002	0.864 ± 0.018	0.443 ± 0.017	0.445 ± 0.011	0.450±0.006	0.225 ± 0.001	69.26±0.73	56.30 ± 1.39
GAT	0.249±0.001	1.201 ± 0.005	0.101±0.001	0.740 ± 0.002	0.196±0.013	0.264 ± 0.002	62.44±0.01	47.79 ± 0.42
NeuralFP	0.349±0.049	1.016 ± 0.081	0.373±0.023	0.484 ± 0.020	0.171±0.007	0.263 ± 0.001	78.74±0.24	62.72 ± 1.91
AttentiveFP	0.180±0.006	1.285 ± 0.001	0.013±0.004	0.763 ± 0.003	0.058±0.011	0.262 ± 0.007	60.56 ± 0.74	39.21 ± 0.66
MPNN	0.249±0.002	1.275 ± 0.011	0.118±0.061	0.732 ± 0.006	0.068±0.015	0.262 ± 0.003	62.53±0.31	40.56 ± 5.46
PAGTN	0.139±0.014	1.255 ± 0.040	0.088±0.194	0.727 ± 0.011	0.092±0.018	0.255 ± 0.007	61.33±0.91	46.52 ± 4.13
Graphormer	0.058±0.015	1.201 ± 0.032	OOM	OOM	0.067±0.046	0.287 ± 0.007	OOM	OOM

ESM-1b	0.562 ± 0.003	0.548 ± 0.004	0.682 ± 0.077	0.282 ± 0.010	0.664 ± 0.002	0.171 ± 0.001	Length Exceeded	Length Exceeded
ESM-2-650M	0.548 ± 0.004	0.563 ± 0.002	0.689 ± 0.020	0.273 ± 0.014	0.694 ± 0.001	0.154 ± 0.001	71.53 ± 0.58	63.33 ± 3.68
Prot-T5-XL	0.553 ± 0.003	0.535 ± 0.006	0.767±0.008	**0.272** ± **0.030**	0.756 ± 0.003	0.136 ± 0.003	74.95 ± 1.01	62.75 ± 0.76
ProtBert	0.566 ± 0.001	0.583 ± 0.007	0.646 ± 0.064	0.322 ± 0.026	0.572 ± 0.001	0.207 ± 0.001	70.70 ± 0.47	62.53 ± 2.67
Deep Prot-T5	0.614	0.642	0.725	0.551	0.874**	0.084**	77.00	68.25**

ChemLLM-7B	−0.019	19.878	−0.187	46273.897	0.017	111.76	50.65	1.64
LlaSMol-Mistral-7B	NaN	2.083	0.094	1.359	NaN	0.268	50.30	6.89

a The ↑ symbol indicates that higher values are better for the corresponding metric. For each method, we used five different random seeds to perform independent runs, reporting the average results along with their standard deviations. On each task, the best method is **bolded**, and the second (or the third closest to the second) best is underlined. We use “**” to denote the method that achieves statistically better results than all the other methods (through statistical tests). These metrics also applied to Table 3-7.

**Table 3.** Results of protein localization prediction.

Model	Subcellular		Binary	
	Acc ↑	Averaged F1 ↑	PR-AUC ↑	Averaged F1 ↑
No. train/valid/test	8945/2248/2768		5161/1727/1746	
CNN	50.18 ± 1.21	30.44 ± 0.28	90.88±0.31	87.74 ± 0.08
CNN-RNN	52.58 ± 0.11	38.21 ± 0.57	91.43 ± 0.45	88.56 ± 1.03
Transformer	42.63±0.68	25.46 ± 0.01	78.38±0.25	72.20 ± 2.21

GCN	47.45±0.47	34.88 ± 0.79	83.43±0.10	81.80 ± 0.07
GAT	45.14±0.10	27.03 ± 0.47	82.15±0.41	81.89 ± 0.19
NeuralFP	45.20±0.49	27.07 ± 0.93	81.14±0.06	79.95 ± 0.09
AttentiveFP	42.38±1.25	23.50 ± 1.34	80.58±0.30	80.08 ± 0.39

ESM-2-650M	79.07 ± 0.05	66.66 ± 1.14	96.63 ± 0.18	91.76 ± 0.47
Prot-T5-XL	80.67 ± 0.04	69.86 ± 0.39	**97.03** ± **0.13**	**93.48** ± **0.07**
LlaSMol-Mistral-7B	15.65	4.99	57.52	0.00
ChemLLM-7B	6.24	0.59	56.32	1.43
Deep Prot-T5	**82.69**	**82.52**	92.17	92.18

**Table 4.** Results of PPI.

Model	PPI Affinity		Yeast PPI		Human PPI	
	$R^{2}$ ↑	MAE ↓	PR-AUC ↑	Averaged F1 ↑	PR-AUC ↑	Averaged F1 ↑
No. of train/valid/test	2127/212/343		1668/131/373		6844/277/227	
CNN	0.646±0.003	**1.764** ± **0.051**	51.93±0.92	25.90 ± 0.10	70.37±1.22	69.65 ± 1.63
CNN-RNN	0.584 ± 0.026	1.886 ± 0.108	53.28±0.85	47.20 ± 3.62	70.45±2.68	69.87 ± 2.59
Transformer	0.425±0.021	2.081 ± 0.133	53.79±1.07	51.93 ± 0.40	59.36±4.00	68.64 ± 1.06

GCN	0.366±0.034	2.443 ± 0.036	58.98 ± 0.72	48.13 ± 3.88	82.21 ± 1.13	71.05 ± 2.90
GAT	0.230±0.001	2.463 ± 0.015	53.72±0.39	57.00 ± 3.83	77.63±3.13	73.92 ± 3.50
NeuralFP	0.100±0.054	2.555 ± 0.040	57.00±1.51	58.94 ± 4.74	80.11±1.25	67.62 ± 1.03
ESM-2-650M	0.592 ± 0.001	1.893 ± 0.005	67.36 ± 0.80	63.99 ± 1.00	**96.17** ± **0.18**	**87.63** ± **0.60**
Prot-T5-XL	0.573 ± 0.011	1.979 ± 0.007	**69.84** ± **0.46**	**66.97** ± **0.01**	95.36 ± 0.07	**87.64** ± **0.98**
LlaSMol-Mistral-7B	−0.008	20.335	53.64	51.39	49.48	49.15
ChemLLM-7B	0.082	28.771	53.65	15.45	47.15	15.49
Deep Prot-T5	0.643	1.870	63.20	**66.70**	76.37	80.28

**Table 5.** Results of epitope and paratope prediction (*residue-level classification*).^a^

Model	IEDB		PDB-Jespersen		SAbDab-Liberis	
	ROC-AUC ↑	Averaged F1 ↑	ROC-AUC ↑	Averaged F1 ↑	ROC-AUC ↑	Averaged F1 ↑
No. of train/valid/test	2211/316/632		313/45/89		716/102/205	
CNN	54.03±0.02	10.34 ± 0.02	**74.46** ± 0.21**	**52.43** ± **0.35**	90.85 ± 0.08	61.51 ± 0.11
CNN-RNN	55.47±0.23	10.96 ± 0.26	70.10±0.97	44.82 ± 1.69	**96.75** ± 0.10**	**69.34** ± **0.10**
Transformer	**59.79** ± 0.06**	**16.79** ± **0.07**	60.10±0.32	27.31 ± 0.56	64.77±0.11	26.73 ± 0.15

a Structure-based and pretrained protein language models took large GPU or CPU memory so we disregrad them in residue-level prediction. The same strategy is applied to secondary structure as well.

**Table 6.** Results of antibody developability prediction (TAP and SAbDab-Chen) and CRISPR repair outcome prediction (CRISPR-Leenay).

Model	TAP		SAbDab-Chen		CRISPR-Leenay	
	$R^{2}$ ↑	MAE ↓	$R^{2}$ ↑	MAE ↓	$R^{2}$ ↑	MAE ↓
No. of train/valid/test	169/24/48		1686/241/482		1065/152/304	
CNN	0.469 ± 0.066	3.217±0.026	0.547 ± 0.014	0.219 ± 0.006	**0.781** ± **0.009**	**0.0745** ± **0.0005**
CNN-RNN	**0.972** ± 0.008**	**0.712** ± 0.069**	0.486 ± 0.012	0.226±0.001	0.771 ± 0.004	0.0755±0.0010
Transformer	0.030 ± 0.011	3.476±0.004	0.452 ± 0.011	0.238±0.012	0.200 ± 0.054	0.1216±0.0020

GCN	0.614 ± 0.054	2.761±0.155	0.434 ± 0.030	0.326±0.015	0.066 ± 0.010	0.1274±0.0019
GAT	0.777 ± 0.007	2.675±0.022	0.356 ± 0.015	0.310±0.010	0.054 ± 0.010	0.1232±0.0001
NeuralFP	0.205 ± 0.035	3.436±0.015	0.452 ± 0.027	0.253±0.011	0.177 ± 0.029	0.1243±0.0001
ESM-2-650M	0.866 ± 0.007	2.452 ± 0.027	**0.600** ± **0.001**	0.224 ± 0.001	0.110 ± 0.011	0.1218 ± 0.0010
Prot-T5-XL	0.837 ± 0.010	2.417 ± 0.056	0.596 ± 0.011	0.229 ± 0.003	0.236 ± 0.004	0.1186 ± 0.0006
LlaSMol-Mistral-7B	NaN	48.104	NaN	0.241	NaN	0.7922
ChemLLM-7B	0.099	45.957	−0.006	32.33	0.061	20.0503
Deep Prot-T5	0.758	2.922	0.536	**0.186**	0.753	0.078

**Table 7.** Results of protein folding prediction (*protein-level and residue-level classification*).

Model	Fold		Secondary Structure	
	Acc ↑	Averaged F1 ↑	Acc ↑	Averaged F1 ↑
No. of train/valid/test	12312/736/718		8678/2170/513	
CNN	8.01 ± 0.44	0.47 ± 0.11	99.44 ± 0.00	87.76 ± 0.06
CNN-RNN	7.50 ± 0.01	1.06 ± 0.01	**99.93** ± **0.00**	98.80 ± 0.00
Transformer	5.34 ± 0.24	0.20 ± 0.02	98.64 ± 0.00	49.66 ± 0.00

GCN	8.09 ± 0.36	2.34 ± 0.15	/	/
ESM-2-650M	49.80 ± 0.36	25.55 ± 0.61	/	/
Prot-T5-XL	50.28 ± 0.52	24.24 ± 0.48	/	/
LlaSMol-Mistral-7B	0.00	0.00	/	/
ChemLLM-7B	0.64	0.04	/	/
Deep Prot-T5	74.00**	73.61**	/	/

### 2.2 Cutting-edge deep learning methods

At the core of deep learning lies the artificial neural network, a machine learning technique inspired by the architecture and functionality of the human brain. What distinguishes deep learning from other machine learning approaches is its exceptional ability to recognize and analyze complex, nonlinear patterns in data, leading to enhanced performance and accuracy. Concretely, we incorporate several cutting-edge neural network architectures into two groups: (i) sequential-based learning and (ii) structural-based learning. Detailed model architectures are described as follows:

*Sequential-based learning* It generally takes a sequence as an input and uses one-hot encoding to pre-encode the input characters. Such learning methods include convolutional neural networks, recurrent neural networks, and transformers (Table 8).

**Table 8.** Memory and time usage of different models.

Dataset	Model	Time (hour)	GPU Memory (GB)
Fluorescence	CNN	0.01000	0.78000
Fluorescence	GCN	0.13200	1.00000
Fluorescence	Prot-T5	0.00160	5.20000
Stability	CNN	0.01600	0.84480
Stability	GCN	0.06100	0.73656
Stability	Prot-T5	0.00472	10.19000
Beta	CNN	0.00150	0.84700
Beta	GCN	0.14000	1.07000
Beta	Prot-T5	0.00060	5.20000
Solubility	CNN	0.01300	0.84700
Solubility	GCN	0.48000	2.70200
Solubility	Prot-T5	0.04000	7.33440
SubCellular	CNN	0.00600	2.94500
SubCellular	GCN	0.07000	8.72600
SubCellular	Prot-T5	0.00060	5.71200
SubCellular-Bin	CNN	0.00110	1.82600
SubCellular-Bin	GCN	0.05200	10.75200
SubCellular-Bin	Prot-T5	0.00030	5.62300
PPI_Affinity	CNN	0.00400	1.37000
PPI_Affinity	GCN	0.03000	1.64600
PPI_Affinity	Prot-T5	0.00050	5.16200
Yeast-PPI	CNN	0.01000	2.69000
Yeast-PPI	GCN	0.32000	12.62000
Yeast-PPI	Prot-T5	0.00020	9.04000
Human-PPI	CNN	0.14000	0.96000
Human-PPI	GCN	1.10000	2.13000
Human-PPI	Prot-T5	0.00320	9.38000
TAP	CNN	0.01000	1.95000
TAP	GCN	0.00300	0.69100
TAP	Prot-T5	0.00030	5.16000
SAbDab-Chen	CNN	0.00300	0.65000
SAbDab-Chen	GCN	0.01000	1.05000
SAbDab-Chen	Prot-T5	0.00060	5.16000
CRISPR	CNN	0.00020	0.62900
CRISPR	GCN	0.00100	0.53800
CRISPR	Prot-T5	0.00050	5.16000
Fold	CNN	0.01000	0.92200
Fold	GCN	0.06000	1.80700
Fold	Prot-T5	0.00020	5.22200

**Figure 2.**
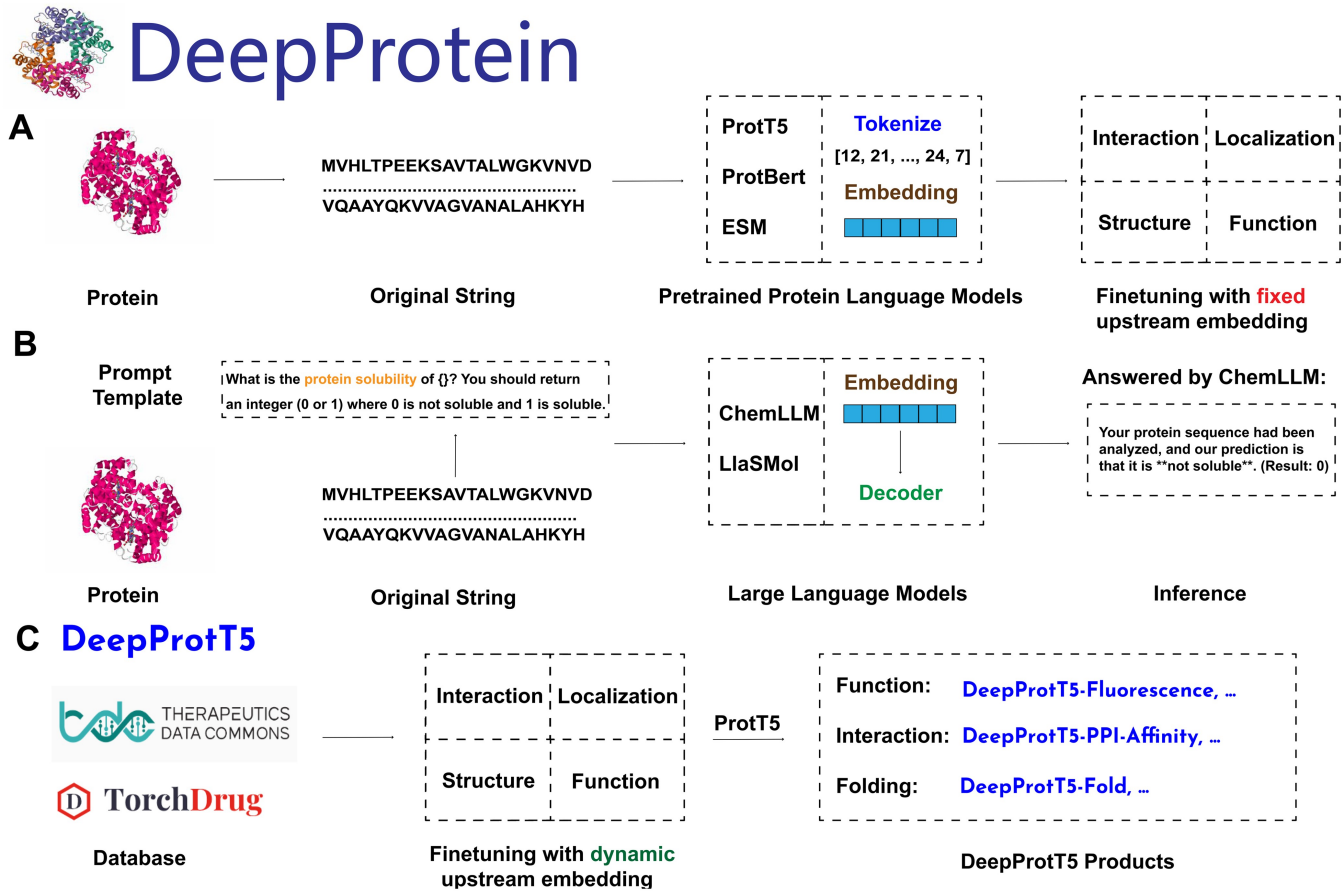
DeepProtein framework, and DeepProt-T5. Part 2. (A) For pre-trained protein language models, we directly use the initial string as the input instead of the transformed SMILES, since most of the protein language models have learned such representations. Strings are tokenized and carried as inputs to the model. We fine-tuned the models on the downstream tasks with the *fixed* embeddings, which means that after feature extraction, the upstream model parameters will not be trained anymore. (B) For large language models, such as ChemLLM and LlaSMol, we did not fine-tune them on the downstream tasks due to limited GPU resources. Instead we directly performed inference on downstream tasks where appropriate prompt templates should be carefully designed here. (C) We extend the family of Prot-T5 to our DeepProt-T5, where the upstream embeddings are dynamic, by using the huggingface trainer. All fine-tuned models could be found here: https://huggingface.co/collections/jiaxie/protlm.

- **Convolutional neural network (CNN)** captures the local patterns in the data features, commonly used to analyze images and text. **(1D) Convolutional neural network (CNN)** takes amino acid sequences as the input. CNN has four layers; the number of filters for the four layers is 32, 64, and 96, respectively. The kernel sizes are 4, 8, and 12, respectively. The convolutional layer is followed by a one-layer MLP (multi-layer perceptron) to predict as a scalar.
- **Recurrent neural network (RNN)** models sequence data and captures the long-term dependencies in the sequence data. RNN has two well-known variants: long short-term memory networks (LSTMs) (Hochreiter and Schmidhuber 1996) and gated recurrent units (GRU) (Cho *et al.* 2014). The difference between GRU and LSTM is that GRU simplifies LSTM by removing the cell state and reducing the number of gates. We use a two-layer bi-directional GRU following three-layer CNN as the neural network architecture. The dimension of the hidden state in GRU is set to 64. ReLU function is applied after each GRU or CNN layer.
- **Transformer** (Vaswani *et al.* 2017) architecture leverages the power of self-attention mechanisms and parallel computation to enhance the neural network’s capability and efficiency in handling sequence data. We use the transformer encoder to represent the amino acid sequence. Two layers of transformer architectures are stacked. The dimension of embedding in the transformer is set to 64. The number of attention heads is set to 4. The ReLU function is applied after each self-attention layer. LayerNorm is applied after MLP layers.
*Structural-based learning* It generally transforms the input sequence into a valid SMILES string, then transforms the chemical substance into a graph. Then, graph filters are learned toward the input graph signal. Such learning methods are widely called Graph Neural Networks. Recently, graph transformers have shown their power in protein function prediction, and we included them as a part of structural-based learning.
- **Graph neural network (GNN)** is a neural network architecture designed to process graph-structured data that takes input from nodes and edges, facilitating the flow of information between connected components to capture their interactions. It learns vector representations for both individual graph nodes and the overall graph structure. We consider the following GNN variants:
  - **Graph convolutional network (GCN)** (Kipf and Welling 2016). GCN is a GNN variant that iteratively updates the node representation by aggregating the information from its neighbors. GCN has three layers, and the node embedding dimension is set to 64. After GCN, all the node embeddings are aggregated with a readout function (Weighted Sum and Max) to get graph-level embedding, followed by a one-layer MLP to get the final prediction. BatchNorm is applied after MLP layers.
  - **Graph attention network (GAT)** (Velickovic *et al.* 2018). GAT uses an attention mechanism to introduce anisotropy into the neighborhood aggregation function. This network features a multi-headed architecture that enhances its learning capacity. The node embedding dimension is 64. Readout function is the same as the one deployed in GCN model.
  - **Message passing neural network (MPNN)** (Gilmer *et al.* 2017). MPNN is a GNN variant that considers passing messages (and modeling interactions) between both edges and nodes based on their neighbors. Edge features are included necessarily compared with GCN and GAT. Readout function is Sum And Max. Node and edge embedding dimension is 64.
  - **Neural fingerprint (NeuralFP)** (Duvenaud *et al.* 2015). NeuralFP uses Graph convolutional network (GCN) (Kipf and Welling 2016) to learn a neural network-based molecular embedding (also known as molecular *neural fingerprint*, or NeuralFP) from a large amount of molecule data without labels. The neural fingerprint is essentially a real-valued vector, also known as embedding. Then, the neural fingerprint is fixed and fed into a three-layer MLP to make the prediction. Node embedding dimension is 64. BatchNorm is applied after MLP layers.
  - **Attentive fingerprint (AttentiveFP)** (Xiong *et al.* 2020). AttentiveFP is a variant of graph neural networks that is enhanced by the attention mechanism when evaluating node and edge embedding. The model consists of three AttentiveFP layers with individual readout function: AttentiveFP readout. Node and edge embedding dimension is 64.
- **Graph transformer** (Yun *et al.* 2019) is a type of neural network architecture designed to process graph-structured data by leveraging self-attention mechanisms. They extend the principles of traditional transformers, enabling them to capture the relationships and interactions between nodes in a graph effectively.
  - **Path-augmented graph transformer** (**PAGTN**) (Chen *et al.* 2019). It used augmented path features to capture long-range (>1 hop) graph properties. The model consists of 5 PAGTN layers with LeakyReLU activation. Node embedding dimension is 64.
  - **Graphormer** (Ying *et al.* 2021). It utilized transformer on graphs with spatial, centrality, and edge encoding. For simplicity and scalability on large graphs, we only deployed one Graphormer layer with ReLU activation. Node embedding dimension is 64. LayerNorm is applied after MLP layers.
- **Foundation model**. A foundation model is a large-scale, pre-trained machine learning model trained on extensive and diverse datasets, typically using self-supervised or unsupervised learning techniques. These models learn generalizable features and patterns from data, allowing them to perform various downstream tasks with minimal task-specific fine-tuning.
  - **ESM**. The Evolutionary Scale Modeling (ESM) utilizes large-scale pretraining on vast protein sequence data to capture evolutionary relationships and functional patterns within proteins (Lin *et al.* 2023, Rives *et al.* 2021). It benefits from Masked Language Modeling (MLM) and the transformer architecture. In this article, we consider two ESM variants with different model sizes: ESM-1b and ESM-2-650M. The latter incorporates Rotary Position Embedding (RoPE) within the ESM-1 framework. We evaluate both models, where the embedding size is 1280.
  - **Prot-T5-XL**. First introduced in ProtTrans (Elnaggar *et al.* 2021), Prot-T5-XL-UniRef50 is based on the T5-3B model and was pre-trained on a large corpus of protein sequences using a self-supervised approach. A key difference from the original T5 model is the denoising objective: while the original T5-3B model used a span denoising objective, this model uses a BART-like MLM denoising objective. The masking probability follows the original T5 training, randomly masking 15% of the amino acids in the input. The embedding dimension is 1024.
  - **ProtBert**. In addition to Prot-T5, ProtTrans includes a model pre-trained on BERT. Bidirectional Encoder Representations from Transformers (BERT) is a transformer-based neural network architecture pre-trained on unlabeled sequence data (Devlin *et al.* 2019). A key difference between ProtBert and the original BERT is how sequences are treated as separate documents, eliminating the need for next sentence prediction. The masking strategy follows the original BERT training, where 15% of the amino acids in the input are randomly masked. The embedding dimension is 1024.
- **Large language model**. In this article, we distinguish foundation models as a class of pre-trained protein language models, whereas we define large language models (LLMs) as decoder-only models designed to generate sequential responses regarding the properties of one or multiple proteins given their sequences and a specific prompt. Due to computational constraints, fine-tuning 7B-scale models is resource-intensive; therefore, we focus on evaluating their performance instead. We consider two protein LLMs in our study: ChemLLM-7B and LlaSMol-Mistral-7B. Their generalization ability to protein-related tasks has not been explored before. Additionally, we provide the chat and prompt template in the appendix.
  - **ChemLLM-7B**. The backbone of ChemLLM-7B (Zhang *et al.* 2024) is the InternLM2-Base-7B model. It was initially trained on the Multi-Corpus-1.7M dataset on Hugging Face and later fine-tuned using instruction-tuning methods on ChemData (7M) and Multi-Corpus (1.7M). ChemLLM-7B has demonstrated superior performance over GPT-4 in retrosynthesis and temperature prediction tasks. The model provides a set of predefined instruction-following templates, which are used in our study and detailed in the appendix.
  - **LlaSMol-Mistral-7B**. The backbone of LlaSMol-Mistral-7B (Yu *et al.* 2024) is Mistral-7B. It was fine-tuned using SMolInstruct, a large-scale, high-quality dataset designed for instruction tuning. SMolInstruct comprises 14 selected chemistry-related tasks and over three million samples, providing a solid foundation for training and evaluating LLMs in the field of chemistry. Specifically, in our experiments, we wrap the input SMILES sequences with 〈PROTEIN〉〈/PROTEIN〉 token pairs to adapt them for protein-related tasks. The template is detailed in the appendix.
- Deep Prot-T5. We have trained Prot-T5-XL on our benchmark datasets individually (Fig. 2). Different from the fixed embeddings, dynamic embeddings enabled us to finetuned the upstream architectures while also maintain a good predictive power for downstream tasks. This leads to a series of DeepProt-T5 models.

*Training setup.* For all models, the maximal training epoch number is set to 100. We used Adam optimizer (Kingma and Ba 2014) for training, with a default learning rate of 0.0001 for sequence-based learning and 0.00001 for structural-based learning. The batch size is equal to 32. More detailed hyper-parameter setups are listed in Table 9 in the appendix. For DeepProt-T5 models, fine-tuning has used a more generalized learning rate of 0.00002 for all models, batch size is equal to 10 to avoid memory errors.

**Table 9.** Default model configurations for protein sequence learning.

Model	lr	Dropout	Activation	No. of heads	No. of layers	Hidden dim	Pooling	Batch size	No. of epochs	Norm
CNN	$10^{-4}$	0.1	ReLU		3	256	MaxPool1d	32	100	
CNN-GRU	$10^{-4}$	0.1	ReLU		2	64		32	100	
Transformer	$10^{-4}$	0.1	ReLU	4	2	64		32	100	LayerNorm
GCN	$10^{-5}$	0.1	ReLU		3	64	Weighted Sum + Max	32	100	BatchNorm
GAT	$10^{-5}$	0.1	ReLU		3	64	Weighted Sum + Max	32	100	
NeuralFP	$10^{-5}$	0.1	ReLU		3	64	Sum + Max	32	100	BatchNorm
AttentiveFP	$10^{-5}$	0.1	ReLU		3	64	AttentiveFPReadout	32	100	
MPNN	$10^{-5}$	0.1	ReLU		6	64	Sum + Max	32	100	
PAGTN	$10^{-5}$	0.1	LeakyReLU		5	64	Weighted Sum + Max	32	100	
Graphormer	$10^{-5}$	0.1	ReLU	8	1	64	MaxPooling	32	100	LayerNorm

### 2.3 Experimental setup and implementation details

*Code base.* This library is an extension of the well-established drug discovery library, DeepPurpose (Huang *et al.* 2021a,b), building upon its foundational capabilities to offer enhanced features for protein-related tasks. By leveraging the strengths of DeepPurpose, this new library provides additional tools and functionalities tailored specifically for protein science. The library is publicly available at https://github.com/jiaqingxie/DeepProtein/.

*Hardware configuration.* All experiments that are mentioned in this article were trained on a 40GB NVIDIA A40 and a 24GB NVIDIA RTX 3090. For DeepProt-T5 fine-tuning, two 24GB NVIDIA RTX 3090 were used. The parameters we provide have ensured the scalable training on these two types of GPUs. When running GNNs on protein localization tasks, we observed a large portion of GPU memory occupied irregularly, so we recommend cutting down the size of the number of workers from 8 to 4 or batch size from 32 to 8 or even smaller to potentially avoid GPU out-of-memory (OOM) problems.

*Software configuration.* The library is implemented in Python 3.9, PyTorch 2.3.0, PyTDC 0.4.1 (Huang *et al.* 2021a,b), DeepPurpose 0.1.5 (Huang *et al.* 2021a,b), and RDKit 2023.9.6 (Bento *et al*. 2020), scikit-learn 1.2.2 (Pedregosa *et al.* 2011), and DGLlife 0.3.2 (Li *et al.* 2021b). Besides, wandb is included in DeepProtein so that researchers can observe the visualization of training curves and test results easily. More details about environment setup could be found in the GitHub.

## 3 Results

For each method, we used three different random seeds to conduct independent runs and reported the average results and their standard deviations. The results of protein function prediction are reported in Tables 2 and 3. The results of PPI are reported in Table 4. The results of epitope and paratope interaction are reported in Table 5. The results of antibody developability prediction are reported in Table 6. The results of protein structure prediction are reported in Table 7.

**Figure 3.**
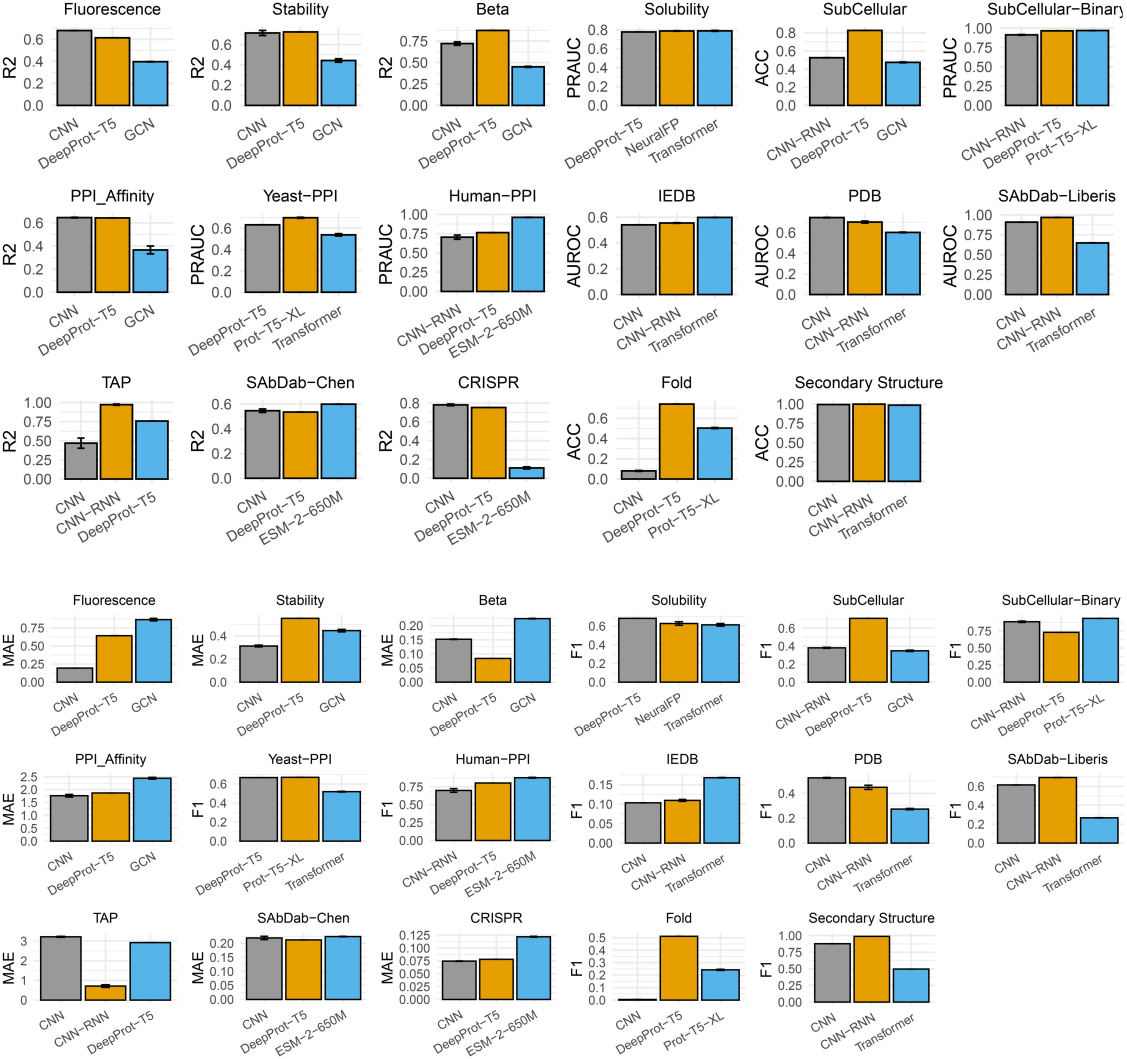
Results of two metrics for selected deep learning methods for DeepProtein Benchmark. For regression task, metrics are Spearman (Pearson) Coefficient and Mean Absolute Error (MAE). For the binary classification task, metrics are ROC (or PR-AUC) and averaged macro F1. For multi classification task, metrics are the accuracy and averaged macro F1. Our DeepProt-T5 are competitive among deep learning methods included in our benchmark, and have improved original Prot-T5 models on six tasks: Beta-lactamase, Solubility, SubCellular, PPI_Affinity, CRISPR, and Fold.

*Statistical test.* We also conduct statistical tests to confirm the superiority of the best-performed method compared with the second-best baseline method. The hypothesis is that the accuracies of the best method are the same as those of the baseline method. Student’s *T*-test is used with significance level alpha as 1% to calculate the *P*-values. When the *P*-values are below the 0.05 threshold, we reject the hypothesis and accept the alternative hypothesis, i.e. the best method is statistically significant compared with the second-best method. We use “**” to denote the method that achieves statistically better results than all the other methods (pass statistical tests) (Fig. 3).

*Key observations*. We summarize the following key observations as takeaways.

**Figure 4.**
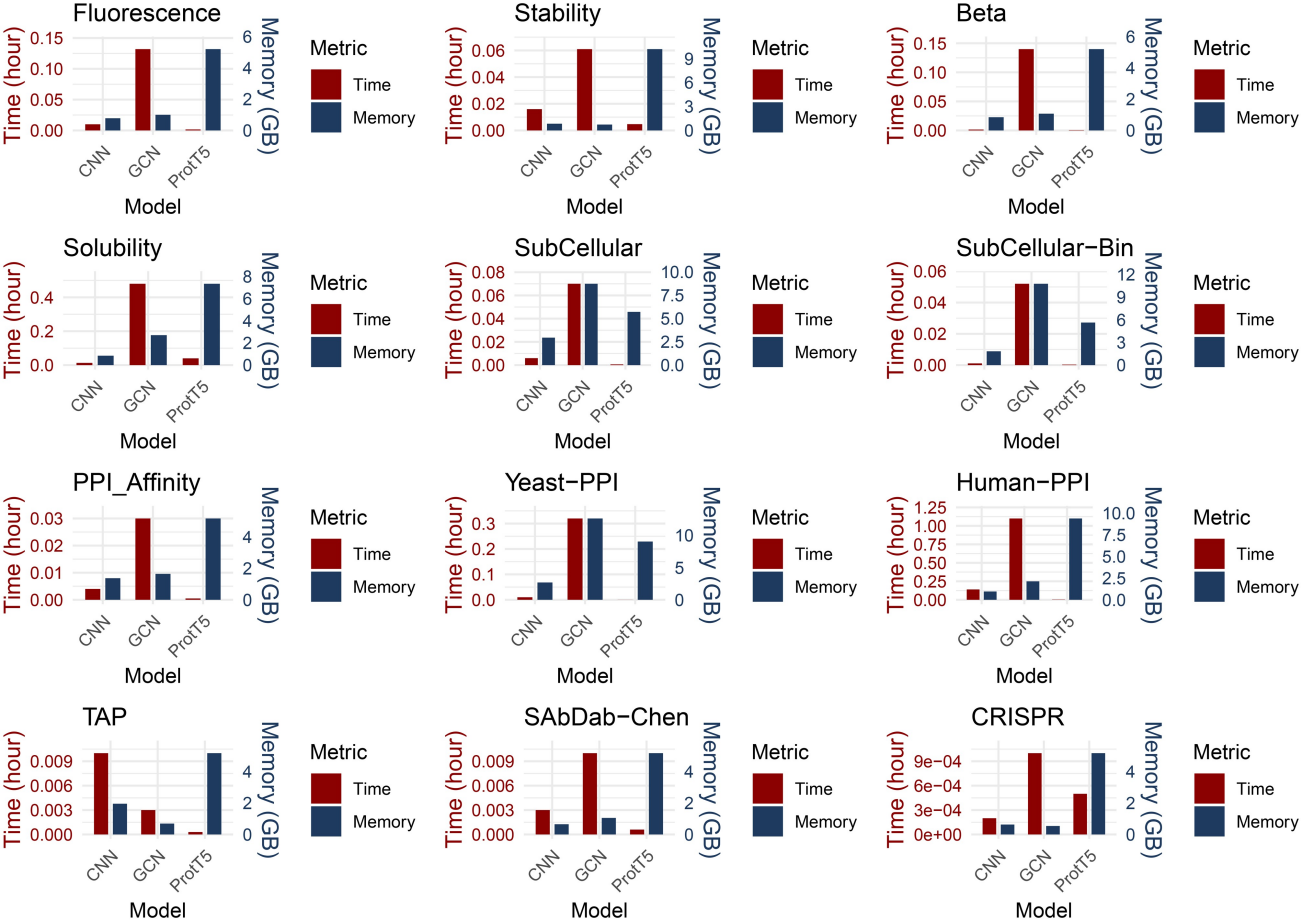
Training Loss of selected datasets: PPI_Affinity, SAbDab_Chen, TAP, and SubCellular.

**Figure 5.**
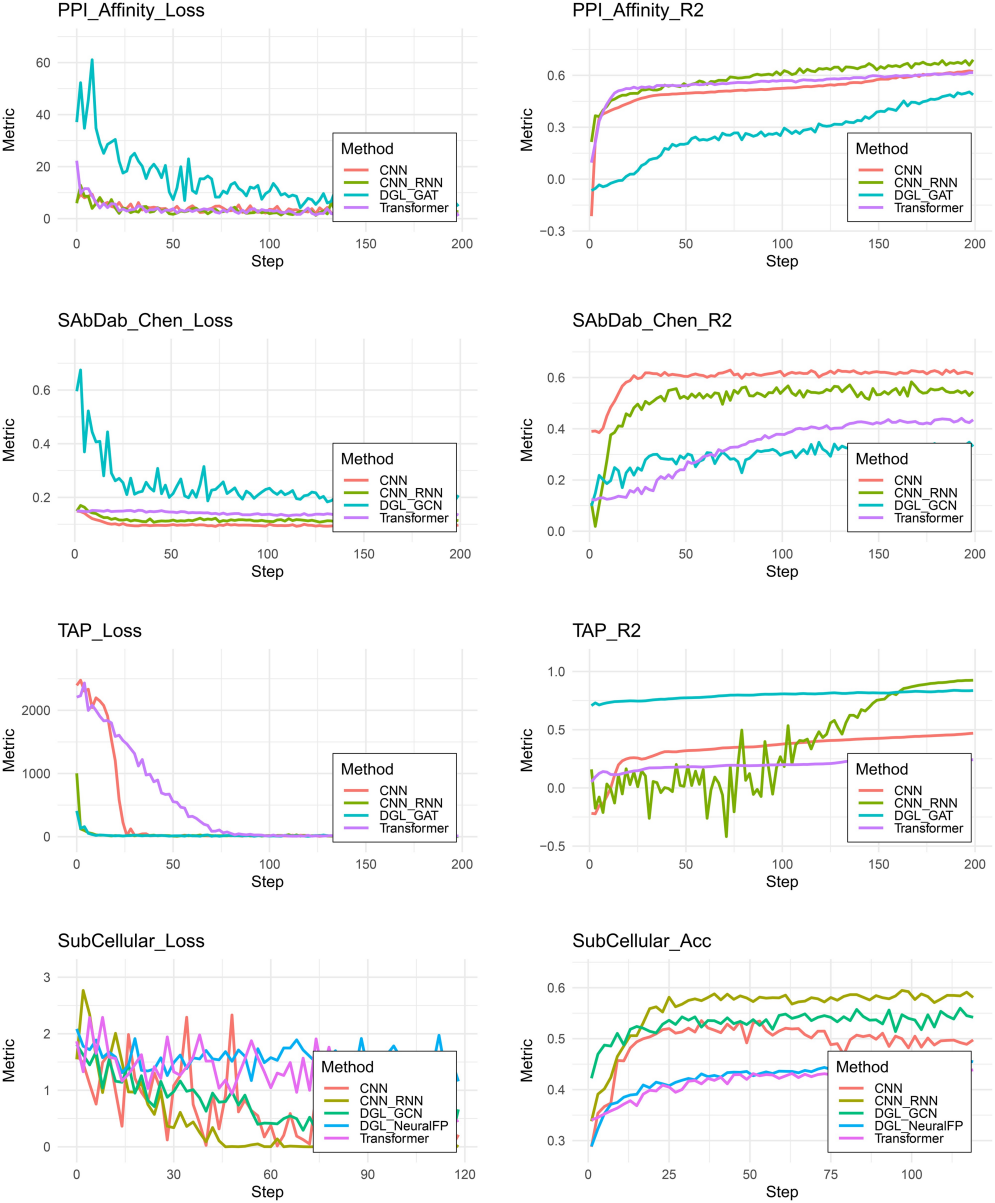
We recorded training time and GPU memory assumptions for each task and each model. Specifically, we extract three representative methods: CNN, GCN, and Prot-T5 from sequence-based, structure-based and pre-trained protein language models. We observed that Prot-T5 and GCN took up more GPU memory than CNN. Prot-T5, where upstream embeddings are fixed, is more efficient in training downstream tasks. Training a GCN model took more time than training a CNN or a Prot-T5 model.

- pre-trained protein language models and our DeepProt-T5 are powerful compared with sequence-based and structure-based neural architectures. Sequence-based neural architectures, such as CNN, RNN, and transformer, obtain also superior performance in most protein sequence learning tasks. Specifically, in 12 out of all the 17 tasks across various protein sequence learning tasks, both sequenced-based models (CNN, RNN, Transformer) and the pre-trained protein language models (Prot-T5-XL, ESM-2-650M and DeepProt-T5) takes the top-2 position.
- Among all the 13 GNN-solvable tasks (except residue-level classification), graph neural networks (GNN) obtain the inferior performance compared with sequenced-based and protein language models. The potential reason would be that SMILES or original string did not provide the 3D information (coordinates) about a protein, the graph topology given by edge featurizer is ill-defined in the deep graph library.
- Among all the graph neural networks (GNNs) across the whole 12 GNN-solvable tasks (except residue-level classification), the earliest variant, GCN (Kipf and Welling 2016), achieves the best performance in 9 tasks.
- **Stability.** From the learning curve (Fig. 4), we find that GNN’s training curve is not stable. In contrast, the sequence-based models, including CNN, RNN, and transformer, converge more stably from the learning curve. This can be observed from Fig. 4. On the contrary, training is more stable, fast and accurate for GAT when it comes to TAP dataset.
- **Computational complexity**. The runtime and memory costs are reported in Fig. 5. We find that GNN-based models are typically computationally inefficient. The key reason behind this is that GNN utilizes molecular graph as the feature, where each atom corresponds to a node and each chemical bond corresponds to an edge. While another model, such as CNN, RNN, and transformer, uses amino acid sequences as the input feature.

## 4 Conclusion

In this article, we have developed DeepProtein, which marks a significant advancement in the application of deep learning to protein science, providing researchers with a powerful and flexible tool to tackle various protein-related tasks. By integrating multiple state-of-the-art neural network architectures and offering a comprehensive benchmarking suite, DeepProtein empowers users to explore and optimize their models effectively. The detailed documentation and tutorials further enhance accessibility, promoting widespread adoption and reproducibility in research. As the field of proteomics continues to evolve, DeepProtein stands to contribute substantially to our understanding of protein functions, localization, and interactions, ultimately driving forward discoveries that can impact biotechnology and medicine.

## 5 Evaluation metrics

In this section, we describe the basic evaluation metrics for both classification and regression tasks. In the part optimization flow it would be further detailed on the end to end training flow.

*Classification metrics.* Most classification tasks are binary classification, except subcellular prediction in protein localization prediction, which is a 10-category classification problem, where we use **accuracy (acc)** (the fraction of correctly predicted/classified samples) as the evaluation metric. In binary classification, there are four kinds of test data points based on their ground truth and the model’s prediction,

1. positive sample and is correctly predicted as positive, known as *True Positive (TP)*;
2. negative samples and is wrongly predicted as positive samples, known as *False Positive (FP)*;
3. negative samples and is correctly predicted as negative samples, known as *True Negative (TN)*;
4. positive samples and is wrongly predicted as negative samples, known as *False Negative (FN)*.
5. **Precision**. The precision is the performance of a classifier on the samples that are predicted as positive. It is formally defined as precision=TP/(TP+FP).
6. **Recall**. The recall score measures the performance of the classifier to find all the positive samples. It is formally defined as recall=TP/(TP+FN).
7. **PR-AUC** (Precision-Recall Area Under Curve). The area under the Precision-Recall curve summarizes the trade-off between the true positive rate and the positive predictive value for a predictive model using different probability thresholds.
8. **ROC-AUC** Area Under the Receiver Operating Characteristic Curve summarizes the trade-off between the true positive rate and the false positive rate for a predictive model using different probability thresholds. ROC-AUC is also known as the Area Under the Receiver Operating Characteristic curve (AUROC) in some literature.

For all these metrics, the numerical values range from 0 to 1, a higher value represents better performance.

*Regression metrics.* In the regression task, both ground truth and prediction are continuous values.

- **Mean squared error (MSE)** measures the average of the squares of the difference between the forecasted value and the actual value. It is defined as $\text{MSE}=\frac{1}{N}\sum_{i=1}^{N}{\left(y_{i}-{\hat{y}}_{i}\right)}^{2},$ where N is the size of the test set; $y_{i}$ and ${\hat{y}}_{i}$ denote the ground truth and predicted score of the i-th data sample in the test set, respectively. MSE value ranges from 0 to positive infinity. A lower MSE value indicates better performance.
- **Mean absolute error (MAE)** measures the absolute value of the difference between the predicted value and the actual value. It is defined as $\text{MAE}=\frac{1}{N}\sum_{i=1}^{N}|y_{i}-{\hat{y}}_{i}|,$ where N is the size of the test set; $y_{i}$ and ${\hat{y}}_{i}$ denote the ground truth and predicted score of the i-th data sample in the test set, respectively. MAE value ranges from 0 to positive infinity. It emphasizes the ranking order of the prediction instead of the absolute value. A lower MAE value indicates better performance.
- **Spearman rank correlation** (ρ), also known as Spearman’s ρ, is a nonparametric statistical test that measures the association between two ranked variables. A higher ρ value indicates better performance.
- **R-squared (** $R^{2}$ **) score** is defined as the proportion of the variation in the dependent variable that is predictable from the independent variable(s). It is also known as the coefficient of determination in statistics. Higher $R^{2}$ scores indicate better performance.

## 6 Optimization flow

### 6.1 Dataset selection and processing flow

As mentioned in the introduction part and Table 1, previous benchmarks either lack 1) the state-of-the-art deep learning methods 2) the diverse real-world data 3) easy-to-use files for researchers outside the computer science domain to use. Hence we collected the data from two main databases which contain approximately 20+ protein tasks which are enough for downstream testing. From them we deleted the tasks that were related to the drug, especially the task drug–target interaction since the DeepPurpose library supported such functionality. For the datasets in the PEER benchmark, DeepProtein just inherited the functions which transformed the data into standard torch datasets, and for the TDC data they were transformed to the standard torch dataset similarly. When loading the dataset, it will load a pair of (protein sequence, aim) or a triple of (protein sequence 1, protein sequence 2, aim), depending on the task type.

### 6.2 Featurization flow

Since we are talking about training here instead of inference, we ignore the featurization flow of large language models. We mainly consider three types of methods here, which are sequence-based, structure-based and pretraind protein language models.

**Sequence-based** models take the tokenized SMILES string as the input X,


(1) $$\begin{array}{c} \begin{array}{c} \mathrm{CNN}:\;X^{\left(l\right)}=X^{\left(l-1\right)}*W^{\left(l\right)}+b^{\left(l\right)} \end{array}\\ \begin{array}{c} \mathrm{RNN}:\;h_{t}=\sigma (W_{h}X_{t}+U_{h}h_{t-1}+b_{h})\\ \mathrm{Attention}:\;\text{Attention}(Q,K,V)=\text{softmax}\left(\frac{QK^{T}}{\sqrt{d_{k}}}\right)V \end{array} \end{array}$$

In CNN, $W^{\left(l\right)}$ is the weight matrix at layer l which is convoluted by the last hidden layer input $X^{\left(l-1\right)}$ and $b^{\left(l\right)}$ is the bias. The hidden state is decided by $X^{\left(l\right)}$. In RNN, we take each token in X as the input at each time step t, where $W_{h}$ is the weight matrix for the current input token (amino acid) $X_{t}$ and $U_{h}$ is the weight matrix for the last hidden state and $b_{h}$ is the bias. We note that the protein sequence would be long in real-world data, so we truncate them to the maximum length 300, which also avoids memory exploding in RNN. In transformer, for each attention block, we could compute Q, K, V by $W_{Q}X$, $W_{K}X$, and $W_{V}X$, then attention is computed by equation (1). Noted that we could aggregated heads of attention to perform multi-head attention.

**Structure-based** models take the graph G as the input, with node features $H^{\left(0\right)}$ and adjacency matrix A. Edge features could be added well if it’s well prepared by the dataset. Especailly for the 2D protein structure, we could only obtain node features by using the features from CNN for instance. GCN, GAT and Graph Transformer’s forms are given by:


(2) $$\begin{array}{c} \begin{array}{c} \;\;\;\;\mathrm{GCN}:\;\;H^{\left(l+1\right)}=\sigma \left({\tilde{D}}^{-\frac{1}{2}}\tilde{A}{\tilde{D}}^{-\frac{1}{2}}H^{\left(l\right)}W^{\left(l\right)}\right) \end{array}\\ \;\;\;\;\mathrm{GAT}:\;\;h_{i}^{\left(l+1\right)}=\sigma \left(\sum_{j\in N(i)\cup \{i\}}\alpha_{ij}^{\left(l\right)}W^{\left(l\right)}h_{j}^{\left(l\right)}\right),\\ \;\;\;\;\;\;\;\;Q=H^{\left(l\right)}W_{Q},\;K=H^{\left(l\right)}W_{K},\;V=H^{\left(l\right)}W_{V}\\ M\mathrm{PNN}:\;m_{v}^{\left(l\right)}=\sum_{u\in N(v)}M\left(h_{v}^{\left(l\right)},h_{u}^{\left(l\right)},e_{vu}\right),\\ \;\;\;\;\;\;\;\;\;h_{v}^{\left(l+1\right)}=U\left(h_{v}^{\left(l\right)},m_{v}^{\left(l\right)}\right) \end{array}$$

In GCN, $H^{\left(l\right)}$ is the node representation at layer l, and $\tilde{A}$ is the adjacency matrix with a self-loop added. $\tilde{D}$ is the degree matrix corresponding to $\tilde{A}$. $W^{\left(l\right)}$ is the weight matrix at layer l. in GAT, $\alpha_{ij}^{\left(l\right)}$ works as a trainable attention parameter to present the attention between node i and node j at layer l. In a general graph transformer, adjacency matrix is added to the attention term which is different from the vanilla self-attention block. Therefore, the complexity is still $O(n^{2})$ if there are $n^{2}$ nodes in the graph. In the message-passing neural network (MPNN), additional edge information $e_{vu}$ is considered for node v and node u.

For the pre-trained protein language models (PLM), the general form could be written as:


(3) $$X^{\prime }=\mathrm{PLM}(X)$$

where we regard PLM as a white box model. We could get the embedding for the whole protein sequence instead of encoding each amino acid one by one which is more efficient than sequence-based or structure-based encoding.

### 6.3 Training flow

After we obtained features from the featurizer module, we train the downstream tasks with a linear layer with the weight **W** and bias b. We consider five machine learning task types (not referring to protein learning tasks), which are single protein regression, single protein classification, protein pair regression, protein pair classification, and token (residue) level single protein classification. We introduce them one by one.

Single protein regression task is that given a single protein’s representation X, after applying the linear layer, we got a floating-point number $\hat{y}$, so mean squared error loss between the true value $y_{i}$ and predicted value ${\hat{y}}_{i}$ is applied during training. For a single protein classification task, we apply the softmax function after the linear layer to decide its class. Either a binary cross-entropy loss (BCELoss) or a general cross-entropy (CE) loss would be back-propagated during the training:


$$\begin{array}{c} \text{Single}\;\text{protein}\;\text{regression:}\;\hat{y}=WX+b\\ L_{\text{MSE}}=\frac{1}{N}\sum_{i=1}^{N}{\left({\hat{y}}_{i}-y_{i}\right)}^{2}\\ \text{Single}\;\text{protein}\;\text{classification:}\;\hat{y}=\text{Softmax}(WX+b)\\ L_{\text{CE}}=-\sum_{i}y_{i}\;\text{log}({\hat{y}}_{i}) \end{array}$$

Protein pair regression is that the input is a pair of proteins ($X_{i},X_{j}$), and the aim is to predict its affinity or some other related interaction metrics, labeled $y_{ij}$ here. The representation of two proteins is concatenated before it is applied to a linear layer. The predicted value is ${\hat{y}}_{ij}$. MSE loss between ${\hat{y}}_{ij}$ and $y_{ij}$ would be back-propagated. For protein pair classification task, we apply a sigmoid function as all labels are either 0 or 1 in our benchmark. BCELoss is being computed.


$$\begin{array}{c} \text{Protein}\;\text{pair}\;\text{regression:}\;{\hat{y}}_{ij}=W(X_{i}||X_{j})+b\\ L_{\text{MSE}}=\frac{1}{N}\sum_{i,j}{\left({\hat{y}}_{ij}-y_{ij}\right)}^{2}\\ \text{Protein}\;\text{pair}\;\text{classification:}\;{\hat{y}}_{ij}=\sigma (W(X_{i}||X_{j})+b)\\ L_{\text{BCE}}=-\sum_{i,j}y_{ij}\;\text{log}({\hat{y}}_{ij})+(1-y_{ij})\;\text{log}(1-{\hat{y}}_{ij}) \end{array}$$

For residue-level single protein classification, we predict the class for each token (amino acid) for each protein sequence, from the token $X_{1}$ to the token $X_{T}$ if the length is equal to T. A softmax is applied here after applying a linear layer and CE-loss is calculated. Note that it is computation inefficient to perform residue level prediction when applying graph neural networks and protein language models and could easily reach memory bound so in our benchmark we only tested those datasets with CNN, CNN-RNN and Transformer architectures. ${\hat{Y}}_{t,c}$ is the probability that $X_{t}$ is being assigned to class c.


$$\begin{array}{c} \text{Residue-level}\;\text{single}\;\text{protein}\;\text{classification:}\;\\ {\hat{Y}}_{t}=\text{Softmax}(WX_{t}+b)\\ L_{\text{CE}}=-\frac{1}{T}\sum_{t=1}^{T}\sum_{c}Y_{t,c}\;\text{log}({\hat{Y}}_{t,c}) \end{array}$$

## 7 Tables of time and memory usage

### 7.1 Prompt template

Template for ChemLLM-7B:

**System Instruction:** 

You are an AI assistant specializing in protein property prediction. Follow the given instruction format.

**User Prompt Format:** 

< |im_start| > user

What is the {protein_property} of the given protein sequence {protein_sequence}? {instruction}

< |im_end| >

< |im_start| > assistant

Template for LlaSMol-Mistral-7B:

What is the {protein_property} of the given protein sequence 〈PROTEIN〉 {protein_sequence} 〈\PROTEIN〉? {instruction}

Instruction and property (task) for each dataset.

**Table:** 

Datasets	Instruction	Property
Fluorescence	You should return a floating-point number.	Fluorescence intensity
Beta	You should return a floating-point number.	Increased activity
Stability	You should return a floating-point number.	Protein stability
Solubility	You should return an integer (0 or 1) where 0 is not soluble and 1 is soluble.	Protein solubility
Subcellular	You should choose an integer within the range [0, 9] to indicate the protein’s location.	Location
Subcellular_Binary	You should return an integer (0 or 1) where 0 is membrane-bound and 1 is soluble.	Location
Tap	You should return a floating-point number.	Developability
SAbDab_Chen	You should return a floating-point number.	Developability
CRISPR	You should return a floating-point number.	Repair outcome
PPI-Affinity	You should return a floating-point number.	Activity of PPI
Yeast-PPI	You should return an integer (0 or 1) where 0 is weak and 1 is strong.	Activity of PPI
Human-PPI	You should return an integer (0 or 1) where 0 is weak and 1 is strong.	Activity of PPI
Fold	You should return an integer within the range [0, 1194].	Global structural topology of a protein on the fold level
Secondary	You should return an integer within the range [0, 2].	Local structures of protein residues in their natural state

## 8 Hyperparameter settings

In Table 9, we have listed a common settings of hyperparameter used in this library. In terms of learning rate (lr), a higher learning rate which is equal to 0.0001 for graph neural networks would lead to failure in training. For Subcellular and its binary version, a training epoch of 60 is enough for convergence. For small-scale protein datasets such as IEDB (Yi *et al.* 2018), PDB-Jespersen, and SAbDab-Liberis, a larger learning rate of 0.001 also leads to convergence and the same performance when using CNN, CNN-RNN, and Transformer. For TAP, SAbDab-Chen and CRISPR-Leenay, larger learning rate of 0.0001 is suggested when training graph neural networks.

## Code availability

Code and data is available at https://github.com/jiaqingxie/DeepProtein
